# Are adolescents with high socioeconomic status more likely to engage in alcohol and illicit drug use in early adulthood?

**DOI:** 10.1186/1747-597X-5-19

**Published:** 2010-08-05

**Authors:** Jennifer L Humensky

**Affiliations:** 1 Edward Hines Jr VA Hospital, Center for Management of Complex Chronic Care, 5000 S. Fifth Avenue (151H), Bldg 1, B251, Hines, IL 60141, USA

## Abstract

**Background:**

Previous literature has shown a divergence by age in the relationship between socioeconomic status (SES) and substance use: adolescents with low SES are more likely to engage in substance use, as are adults with high SES. However, there is growing evidence that adolescents with high SES are also at high risk for substance abuse. The objective of this study is to examine this relationship longitudinally, that is, whether wealthier adolescents are more likely than those with lower SES to engage in substance use in early adulthood.

**Methods:**

The study analyzed data from the National Longitudinal Survey of Adolescent Health (AddHealth), a longitudinal, nationally-representative survey of secondary school students in the United States. Logistic regression models were analyzed examining the relationship between adolescent SES (measured by parental education and income) and substance use in adulthood, controlling for substance use in adolescence and other covariates.

**Results:**

Higher parental education is associated with higher rates of binge drinking, marijuana and cocaine use in early adulthood. Higher parental income is associated with higher rates of binge drinking and marijuana use. No statistically significant results are found for crystal methamphetamine or other drug use. Results are not sensitive to the inclusion of college attendance by young adulthood as a sensitivity analysis. However, when stratifying by race, results are consistent for white non-Hispanics, but no statistically significant results are found for non-whites. This may be a reflection of the smaller sample size of non-whites, but may also reflect that these trends are driven primarily by white non-Hispanics.

**Conclusions:**

Previous research shows numerous problems associated with substance use in young adults, including problems in school, decreased employment, increases in convictions of driving under the influence (DUI) and accidental deaths. Much of the previous literature is focused on lower SES populations. Therefore, it is possible that teachers, parents and school administrators in wealthier schools may not perceive as great to address substance abuse treatment in their schools. This study can inform teachers, parents, school administrators and program officials of the need for addressing drug abuse prevention activities to this population of students.

## Background

The relationship between childhood socioeconomic status (SES) and behavioral health in adulthood has long been of interest to researchers and policymakers. A few studies have found that adolescents with low SES have a greater propensity toward substance use during adolescence. Goodman and Huang [1], studying the first wave of AddHealth data cross-sectionally, found that having low SES was associated with greater alcohol use and with greater cigarette and cocaine use among white teenagers. Goodman and colleagues [2] found that lower household income and parental education were associated with greater adolescent depression. Friestad and colleagues [3] found that low parental education and moderate household income was associated with greater rates of smoking in adolescents. Reinherz and colleagues [4], examining 360 respondents followed from 1977-2000, found that low family SES and larger family size were associated with increased probability of substance abuse disorders in early adulthood. An analysis by Hamilton and colleagues [5], of the Ontario Student Drug Use Survey, found that adolescents (ages 12-19) with college-educated parents were less likely to engage in hazardous or harmful drinking or illicit drug use.

However, there is growing evidence that adolescents with higher SES may also be at risk for developing substance use disorders. There is evidence that substance use in adults, particularly alcohol use, may be sensitive to price, as some studies have shown that consumption decreases as price increases [6-9]. For adolescents with higher SES, having greater financial resources may indicate that the relative cost of substance use, that is the opportunity cost of substance use relative to other consumption, may be lower than for adolescents with lower SES. This is consistent with the usual demand model for goods and services, and could indicate a higher demand among wealthier adolescents. This was found in a 2007 study of British adolescents by Bellis and colleagues, which found that adolescents with more spending money were more likely to drink frequently, binge drink and to drink in public [10], as well as in a study of college students in the United States, which found that college students with lower levels of spending money had lower levels of drinking and getting drunk [11],

Engagement in substance use can have negative implications for young adults. Previous research has shown that substance use at young ages is associated with decreased educational attainment [12,13] and labor market productivity [14,15]. Binge drinking in particular has been linked to driving under the influence of alcohol (DUI) and accidental deaths in college-age students [16]. As illicit drugs are illegal in the United States, the use of these substances places young adults at risk of involvement in the criminal justice system. Thus, substance use can have substantial negative consequences for young adults. However, as much previous literature has focused on the substance use of lower income adolescents [1-5], it is possible that parents, teachers, policymakers and program administrators may be less focused on the possible long-term implications of substance use on adolescents with higher SES [17-19].

The purpose of this study is to examine whether adolescents with high SES, as measured by parental education and income, are more likely to engage in alcohol and illicit drug use in early adulthood, using a prospective, nationally-representative sample of secondary school students in the United States. This expands upon the work of Bellis and colleagues [10], who examined cross-sectional data on alcoholism in the United Kingdom and Martin and colleagues [11], who examined prevalence of alcohol use among college students in the United States. As much of the previous literature has focused on adolescents with lower SES, the findings of this study could help parents, teachers and practitioners to identify students who may be at risk for future substance use and educational problems.

## Methods

### Data

The data comes from the National Longitudinal Survey of Adolescent Health (AddHealth). The first wave of AddHealth was conducted in 1994-1995, and consisted of a nationally representative sample of school students in grades 7-12 in the United States. The sample was a school-based cluster design, which was selected to ensure a nationally representative population, by region, urbanicity, racial and ethnic composition, school type, and school size [20].

A total of 20,745 students completed the in-home interview. Parents of students who were selected for the in-home interview were also interviewed. The third wave of the study, which followed-up respondents from the Wave I interview, was completed in 2001-02, when respondents were 18-27 years old. A few respondents (72 students) with mental retardation were excluded from the sample, as their educational trajectories are likely to differ from those of their peers.

### Estimation Models

The estimation models examine the relationship between adolescent SES and subsequent use of alcohol and illicit drugs, controlling for baseline use of alcohol and illicit drugs and mental health (depressive symptoms, delinquency and suicidality) and a set of other individual, family school and neighborhood characteristics.

The outcome variables of interest are binge drinking and illicit drug use in early adulthood. AddHealth asks respondents separately about their use of alcohol, marijuana, cocaine, crystal methamphetamine and any other drug use. As the use of these substances is measured separately in AddHealth, they are analyzed separately in these analyses. Substance use was measured as a binary indicator of use, as previous research has found that responders tend to report accurately whether they consume substances, but tend to under-report the amount consumed [6]. Sensitivity analyses found that results were qualitatively similar when examining a continuous measure of use (not shown).

The key independent variable was socioeconomic status at baseline, as measured by parental education and income. As these were assessed separately in AddHealth, they are analyzed separately in these analyses as well. The parents' socioeconomic status reflects the economic status of the family in which the adolescent resides at baseline. Previous research has shown that parental socioeconomic status reflects the child's socioeconomic status [21]. The highest educated parent was used rather than the education level of the mother and father to avoid losing respondents living in single parent families [22]. Household income was also included as a separate measure of SES, as household income captures smaller gradations in family well-being than parental income [23].

A number of the adolescent's baseline personal characteristics were also included in the model. The adolescent's baseline binge drinking and illicit drug use was included in the model in order that these models measure change in substance use from baseline to follow-up in early adulthood. In addition, other behavioral health factors at baseline were controlled for, including delinquency, suicidality and depressive symptoms, due to the high rates of comorbidity of mental health diagnoses among persons with substance abuse [24].

A number of demographic variables were also included in the model, including age, gender, and race/ethnicity. Additional individual characteristics that have been shown to be correlated with educational attainment were included, such the Peabody Picture Vocabulary (PPVT) score, which measures cognitive ability [25], and general health, which measures physical health and may account for endogeneity between mental health and labor market outcomes [26,27].

A number of family background variables were also included in the model. An indicator of whether the respondent was the first child born in the family was included, as previous research has shown that children born first in the family tend to have better educational outcomes [28]. Family structure (single parent household, stepfamily and foster/other) is included, as children from non-traditional families often face barriers to schooling [29]. An indicator for whether the respondent's biological mother and father have alcoholism is included as alcoholism has a high genetic component [30].

The estimation model is of the form:

$$\text{Logit}\left({\text{p}}_{\text{i},\text{t}+\text{1}}\right)=\beta_{00}+\beta_{0\text{1}}{\text{SES}}_{\text{i}}+\beta_{0\text{2}}{\text{BH}}_{\text{i}}+\beta_{0\text{3}}{\text{X}}_{\text{i}}+\varepsilon_{\text{i}}$$

Where:

p_i,t+1 _= Probability of substance use at Wave III, as measured by binge drinking, marijuana use, cocaine use, crystal methamphetamine use and other drug use, estimated separately. Binge drinking was measured as a binary variable with a positive value indicating 5 or more drinks in one setting more than once a month in the past year. Marijuana, cocaine, crystal methamphetamine and other drug use was measured as a binary variable with a positive value indicating any use in the past 30 days.

SES_i _= Socioeconomic status at baseline, as measured by parental education and household income. Parental education was measured by the education level of the highest educated parent living with the adolescent, and was categorized by the highest educated parent having not completed high school, having completed high school only, having education beyond high school but less than a college degree, or having a college degree. Household income was measured continuously and was topcoded in AddHealth at $999,000, so reported income ranged from $0-$999,000.

BH_i _= A number of behavioral health (mental health and substance use) characteristics at baseline in adolescence (Wave I interview) were included in the model: binge drinking, marijuana, cocaine, inhalant and other drug use at baseline, as well as depressive symptoms, delinquency and suicidality. Each of these components was included separately in the model. As with the outcome variables, binge drinking in adolescence was measured as a binary variable with a positive value indicating 5 or more drinks in one setting more than once a month in the past year. Marijuana, cocaine, inhalant and other drug use was measured as a binary variable with a positive value indicating any use of these substances in the past 30 days. Depressive symptoms are measured by the Center for Epidemiologic Studies Depression scale (CES-D) [31,32]. Delinquency is a scale of 0-12 of criminal behaviors in the past year [33]. Suicidality is measured by a binary variable with a positive value indicating a suicide attempt in the past year.

X_i _= A number of individual and family characteristics included as control variables. These include demographic variables such as gender, age, age squared (to account for non-linearities in age), and race/ethnicity [34]. Race/ethnicity categories are defined as Hispanic or white non-Hispanic, black non-Hispanic, Asian non-Hispanic, or other race, non-Hispanic. The Peabody Picture vocabulary test score is measured on a continuous scale, and is standardized so that the mean is approximately 100. General health is categorized as excellent, very good, good, or fair/poor. Family structure is categorized as two biological parents, single parent household, stepfamily or foster/other family. A binary indicator indicates whether the adolescent is the first born in the family, and whether the adolescent has a biological mother or father with alcoholism.

These equations were analyzed using Stata 10.0. Logit models were analyzed because the outcome variables of interest were dichotomized, and odds ratios are reported in the tables. The research protocol was approved by the University of Chicago Social and Behavioral Sciences Institutional Review Board (#H06238).

### Missing Observations

A total of 20,745 students completed the AddHealth in-home interview, and of these, 15,170 followed up at Wave III. There is thus attrition as well as item non-response. Item non-response is particularly problematic for the items asked of parents, parental income and alcoholism. The final sample, with no missing observations, was 9,872. Therefore, sample loss is a concern. This was addressed using re-weighting and imputation measures. Results were qualitatively similar under re-weighting and imputation, and so the results presented here use the AddHealth's weights and original sample to allow for greater replicability. The full detail is provided in Additional file 1.

## Results

### Descriptive Statistics

Table 1 shows descriptive statistics. In adolescence, the average household income was about $46,000 at the time of baseline data collection (1994-1995). In about 9 percent of households, the highest educated resident parent did not complete high school. In about 34 percent of households, the highest educated resident parent had a college education.

**Table 1:** Descriptive Statistics (n = 9872)

Variable	Mean	St. Error
Baseline Variables, Wave I		
		
Household income, in $1000s, range: 0-999 (topcoded)	46.07	1.70
Highest educated resident parent has less than high school education	9.40%	1.05%
...high school education	25.82%	1.14%
...more than high school education	31.26%	0.97%
...college graduate and beyond	33.53%	1.85%
Binge Drinking, Wave I	16.98%	0.87%
Marijuana Use, Wave I	13.70%	0.80%
Cocaine Use, Wave I	1.10%	0.16%
Inhalant Use, Wave I	1.76%	0.20%
Other Drug Use, Wave I	4.05%	0.39%
CES-D Score, Wave I	11.19	0.16
Delinquency Scale, Wave I	1.59	0.04
Suicidality, Wave I	3.91%	0.29%
Verbal Ability (PPVT)	101.71	0.57
Age at Wave I	15.78	0.12
Male	50.84%	0.76%
White, non-Hispanic	69.02%	2.74%
Black, non Hispanic	13.28%	1.91%
Hispanic	10.63%	1.57%
Asian, non-Hispanic	2.59%	0.63%
Other/multi race	4.48%	0.45%
Excellent general health	27.98%	0.66%
Very good general health	40.52%	0.84%
Good general health	24.93%	0.65%
Fair/poor general health	6.57%	0.45%
Single parent household	25.40%	1.09%
Two biological parent household	58.00%	1.26%
Stepfamily	10.07%	0.47%
Foster/other household	6.56%	0.40%
Biological parent alcoholism	16.05%	0.72%
Firstborn	52.19%	0.97%
		
Outcome Variables, Wave III		
		
Binge Drinking	24.73%	1.04%
Marijuana Use	23.98%	0.80%
Cocaine Use	3.69%	0.30%
Crystal Methamphetamine Use	1.46%	0.18%
Other Drug Use	5.34%	0.38%

Descriptive statistics are weighted, cluster and strata corrected.

About 25 percent engaged in binge drinking in early adulthood (Wave III), about 24 percent engaged in marijuana use, about 4 percent engaged in cocaine use, one percent in crystal methamphetamine use, and about 5 percent in other drug use.

### Regression Results

Table 2 shows the regression results examining the association between SES in adolescence and subsequent substance use in early adulthood. Higher parental education is associated with higher odds of binge drinking, marijuana use and cocaine use. For an individual with a college-educated parent, the odds of binge drinking in early adulthood are 1.458 times as large as the odds for an individual with a high school-educated parent once all controls are included in the model (AOR = 1.458, 95% CI [1.190-1.788]). Likewise, for an individual with a college-educated parent, the odds of engaging in marijuana use in early adulthood are 1.265 times as large as the odds for an individual with a high school-educated parent (AOR = 1.265, 95% CI [1.038-1.541]). The odds of engaging in cocaine use in early adulthood are 1.614 times as large for an individual with a college-educated parent versus a high school-educated parent (AOR = 1.614, 95% CI [1.088-2.395]). No statistically significant effects are found for crystal methamphetamine and other drug use.

**Table 2:** The Relationship between Parental Education in Adolescence and Substance Use in Early Adulthood. (n = 9872)

	Binge Drinking	Marijuana Use	Cocaine Use	Crystal Meth. Use	Other Drug Use
**Parents' Education**					
*Unadjusted OR (95% CI)*					
Highest educated resident parent less than high school	0.631 (0.490-0.812)***	0.737 (0.566-0.961)*	0.851 (0.446-1.622)	0.384 (0.147-1.007)†	0.443 (0.241-0.817)**
...High school	Reference	Reference	Reference	Reference	Reference
...Some college	1.204 (1.000-1.449)†	1.187 (1.016-1.387)*	1.294 (0.870-1.925)	0.914 (0.542-1.541)	1.091 (0.779-1.528)
...College graduate	1.642 (1.335-2.020)***	1.306 (1.091-1.563)**	1.605 (1.117-2.306)*	0.672 (0.375-1.205)	1.203 (0.869-1.665)
Joint test	F(3,126) = 17.63***	F(3,126) = 6.32***	F(3,126) = 2.99*	F(3,126) = 1.72	F(3,126) = 4.19**
					
*Adjusted OR (95% CI)*					
Highest educated resident parent has less than high school	0.812 (0.605-1.089)	0.827 (0.601-1.138)	1.060 (0.508-2.214)	0.417 (0.161-1.081)†	0.525 (0.275-1.002)†
...High school	Reference	Reference	Reference	Reference	Reference
...Some college	1.092 (0.894-1.333)	1.129 (0.956-1.334)	1.225 (0.814-1.844)	0.823 (0.484-1.399)	0.977 (0.694-1.375)
...College graduate	1.458 (1.190-1.788)***	1.265 (1.038-1.541)*	1.614 (1.088-2.395)*	0.643 (0.336-1.233)	1.024 (0.701-1.498)
Joint test of parental education	F(3,126) = 7.72***	F(3,126) = 3.35*	F(3,126) = 2.31†	F(3,126) = 1.40	F(3,126) = 1.73

Statistical significance based on t-tests: † p < .10; * p < .05; ** p < 0.01; *** p < .001

Note: Dependent variables assessed at Wave III. Independent variables assessed at Wave I. Adjusted models control for binge drinking, marijuana use, cocaine use, inhalant use and other drug use at baseline, CESD, delinquency, suicidality, PPVT score, gender, age, age squared, race/ethnicity, general health, family structure (two biological parents, single parent, stepfamily, foster/other), parent alcoholism and whether adolescent is firstborn in family. Logit models with data weighted, clustered and strata corrected.

Other drug use includes LSD, PCP, ecstasy, mushrooms, inhalants, ice, heroin, prescription medicine not prescribed for you or any other drug.

Table 3 shows the regression results examining the association between SES as measured by household income in adolescence and substance use in early adulthood. Higher household income is associated with higher probability of binge drinking and marijuana use. An additional $1000 in annual household income in adolescence is associated with an increase of 1.003 in the odds of binge drinking in early adulthood (AOR = 1.003, 95% CI [1.001-1.004]). It should be noted that this result, while statistically significant, is quite small. Likewise, an additional $1000 in annual income in adolescence is associated with an increase of 1.002 in the odds of marijuana use in early adulthood (AOR = 1.002, 95% CI [1.000-1.003]), and an increase of 1.002 in the odds of cocaine use, though the results for cocaine use lose statistical significance once controls are added to the model (OR = 1.002, 95% CI [1.000-1.004], AOR = 1.002, 95% CI [0.999-1.004]). No statistically significant results are found for crystal methamphetamine or other drug use.

**Table 3:** The Relationship between Household Income in Adolescence and Substance Use in Early Adulthood. (n = 9872)

	Binge Drinking	Marijuana Use	Cocaine Use	Crystal Meth. Use	Other Drug Use
**Household Income**					
*Unadjusted OR (95% CI)*					
Income (1000s)	1.004 (1.003-1.006)***	1.002 (1.001-1.003)**	1.002 (1.000-1.004)*	0.995 (0.988-1.002)	1.001 (1.000-1.002)
					
*Adjusted OR (95% CI)*					
Income (1000s)	1.003 (1.001-1.004)***	1.002 (1.000-1.003)*	1.002 (0.999-1.004)	0.992 (0.983-1.002)	1.000 (0.998-1.002)

Statistical significance based on t-tests: † p < .10; * p < .05; ** p < 0.01; *** p < .001

Note: Dependent variables assessed at Wave III. Independent variables assessed at Wave I. Adjusted models control for binge drinking, marijuana use, cocaine use, inhalant use and other drug use at baseline, CESD, delinquency, suicidality, PPVT score, gender, age, age squared, race/ethnicity, general health, family structure (two biological parents, single parent, stepfamily, foster/other), parent alcoholism and whether adolescent is firstborn in family. Logit models with data weighted, clustered and strata corrected.

Other drug use includes LSD, PCP, ecstasy, mushrooms, inhalants, ice, heroin, prescription medicine not prescribed for you or any other drug.

### Sensitivity Analyses

Tables 4 and 5 show sensitivity analyses. Table 4 shows the results when college attendance in young adulthood (by Wave III) is included in the model. It should be noted that the prospective nature of the model is violated in this sensitivity analysis, as an independent variable, college attendance, is measured at Wave III, as is the outcome variable. Still, this analysis is useful in examining whether the relationships seen in Tables 2 and 3 are simply functions of college attendance. It is possible that wealthier young adults are more likely to attend college, and thus to be living near peers who are engaging in substance use, particularly alcohol and marijuana use. College attendance by Wave III is measured by enrollment or graduation from a two or four year college at the Wave III interview.

**Table 4:** Sensitivity Analysis: The Relationship between Parental Education and Income in Adolescence and Substance Use in Early Adulthood, controlling for College Attendance by Wave III (n = 9872)

	Binge Drinking	Marijuana Use	Cocaine Use	Crystal Meth. Use	Other Drug Use
**Parents' Education**					
*Adjusted OR (95% CI)*					
Highest educated resident parent less than high school	0.958 (0.674-1.362	0.817 (0.558-1.195)	1.496 (0.554-4.038)	0.700 (0.193-2.538)	0.597 (0.298-1.195)
...High school	Reference	Reference	Reference	Reference	Reference
...Some college	1.140 (0.919-1.414)	1.235 (1.034-1.475)*	1.449 (0.855-2.457)	1.138 (0.513-2.527)	0.964 (0.645-1.442)
...College graduate	1.472 (1.189-1.821)***	1.392 (1.117-1.734)**	2.047 (1.203-3.483)**	0.962 (0.419-2.205)	1.115 (0.725-1.714)
Joint test	F(3,125) = 5.44**	F(3,125) = 4.72**	F(3,125) = 2.64†	F(3,125) = 0.22	F(3,125) = 1.31
					
**Household Income (1000s)**					
*Adjusted OR (95% CI)*					
					
Income (1000s)	1.003 (1.000-1.004)**	1.002 (1.000-1.003)*	1.002 (1.000-1.004) †	0.996 (0.988-1.004)	1.000 (0.998-1.002)

Statistical significance based on t-tests: † p < .10; * p < .05; ** p < 0.01; *** p < .001

Note: Dependent variables assessed at Wave III. Independent variables assessed at Wave I. Adjusted models control for college enrolment or graduation, binge drinking, marijuana use, cocaine use, inhalant use and other drug use at baseline, CESD, delinquency, suicidality, PPVT score, gender, age, age squared, race/ethnicity, general health, family structure (two biological parents, single parent, stepfamily, foster/other), parent alcoholism and whether adolescent is firstborn in family. Logit models with data weighted, clustered and strata corrected.

Other drug use includes LSD, PCP, ecstasy, mushrooms, inhalants, ice, heroin, prescription medicine not prescribed for you or any other drug.

**Table 5:** Sensitivity Analysis: The Relationship between Parental Education d in Adolescence and Substance Use in Early Adulthood, by race (White non-Hispanic and Non-white) (n = 9872)

	Binge Drinking	Marijuana Use	Cocaine Use	Crystal Meth. Use	Other Drug Use
**White, Non-Hispanic** **(n = 5580)**					
**Parents' Education**					
*Adjusted OR (95% CI)*					
Highest educated resident parent less than high school	0.938 (0.604-1.458)	0.839 (0.493-1.429)	0.816 (0.300-2.216)	0.482 (0.165-1.414)	0.584 (0.224-1.518)
...High school	Reference	Reference	Reference	Reference	Reference
...Some college	1.180 (0.944-1.474)	1.175 (0.975-1.415) †	1.218 (0.767-1.933)	0.746 (0.399-1.396)	1.047 (0.686-1.597)
...College graduate	1.652 (1.316-2.073)***	1.357 (1.082-1.700)**	1.575 (1.005-2.467)*	0.492 (0.224-1.083) †	1.045 (0.670-1.630)
Joint test	F(3,126) = 7.79***	F(3,126) = 3.31*	F(3,126) = 1.90	F(3,126) = 1.37	F(3,126) = 0.56
					
**Non-White** **(n = 4292)**					
**Parents' Education**					
*Adjusted OR (95% CI)*					
Highest educated resident parent less than high school	0.728 (0.451-1.745)	0.759 (0.509-1.134)	1.663 (0.616-4.493)	0.491 (0.069-3.501)	0.538 (0.260-1.113) †
...High school	Reference	Reference	Reference	Reference	Reference
...Some college	0.912 (0.656-1.268)	1.047 (0.789-1.391)	1.420 (0.598-3.368)	1.608 (0.591-4.374)	0.924 (0.512-1.666)
...College graduate	0.895 (0.605-1.326)	1.001 (0.690-1.452)	1.859 (0.852-4.056)	1.782 (0.768-4.135)	1.011 (0.507-2.013)
Joint test	F(3,126) = 0.58	F(3,126) = 0.91	F(3,126) = 0.94	F(3,126) = 1.09	F(3,126) = 1.17

Statistical significance based on t-tests: † p < .10; * p < .05; ** p < 0.01; *** p < .001

Note: Dependent variables assessed at Wave III. Independent variables assessed at Wave I. Adjusted models control for binge drinking, marijuana use, cocaine use, inhalant use and other drug use at baseline, CESD, delinquency, suicidality, PPVT score, gender, age, age squared, general health, family structure (two biological parents, single parent, stepfamily, foster/other), parent alcoholism and whether adolescent is firstborn in family. Logit models with data weighted, clustered and strata corrected.

Other drug use includes LSD, PCP, ecstasy, mushrooms, inhalants, ice, heroin, prescription medicine not prescribed for you or any other drug.

The results show that the relationships seen above are robust to the inclusion of college attendance. Persons with higher parental education (as measured in adolescence) have a higher probability of binge drinking (joint test p < 0.01), marijuana (joint test p < 0.01) and cocaine use (joint test approaching significance at p < 0.10), when controlling for college attendance. In addition, higher household income in adolescence is associated with a higher probability of binge drinking (AOD = 1.003, 95% CI [1.000-1.004]), marijuana use (AOD = 1.002, 95% CI [1.000-1.003]), and cocaine use (AOD = 1.002, 95% CI [1.000-1.004]) in early adulthood when college attendance is controlled for. No statistically significant results were found for crystal methamphetamine or other drug use. The results of this sensitivity analysis indicate that the higher rates of binge drinking, marijuana and cocaine use are not solely the result of greater college attendance.

Table 5 stratifies results by race, white non-Hispanic versus non-whites. Although results for white non-Hispanics are qualitatively similar to the results in Table 2 for the whole population, none of the results for the non-whites are statistically significant. This may be a function of the smaller sample size (n = 5580 for whites and n = 4292 for non-whites). It may also be that the results found are being driven primarily by white adolescents.

## Discussion

The results from this study indicate that higher SES in adolescence, as measured by parental education and household income in adolescence, is associated with higher rates of substance use, particularly binge drinking, marijuana use and cocaine use, in early adulthood. No statistically significant results were found for crystal methamphetamine or other drug use. Results were consistent when controlling for college attendance by young adulthood as a sensitivity analysis. When stratifying by race, results were consistent for white non-Hispanics, but no significant results were found for non-whites. This may be a function of a smaller sample size of the non-white sample, or it may be that the results are driven primarily by white non-Hispanic respondents. In addition, sensitivity analyses were performed while not controlling for adolescent substance use, and results were qualitatively similar (not shown).

Previous literature has shown that the relationships between SES and substance use vary by age. The results in this paper are somewhat contrary to previous literature in youth which has shown that lower SES is associated with higher rates of substance use problems [1-5]. However, the results of this study are consistent with previous research in adults, which found that demand for illicit substances is price sensitive [6-9], and thus predicts that substance use will increase as income is higher. This therefore indicates that the behavior of young adults more closely reflects that of adults rather than that of youth. The results are also consistent with Bellis and colleagues [10], who found that adolescents with more spending money reported greater substance use and with Martin and colleagues [11], who found that college students with more spending money engaged in greater alcohol use. This study provides additional evidence for these earlier findings in a longitudinal, nationally-representative sample of adolescents in the United States, and in illicit substances in addition to alcohol use. In addition, it is possible that parental education may have a distinct influence on subsequent college attendance by the adolescent, distinct from general socioeconomic status. Parents with higher education may have a greater influence on their adolescent's choice to attend college, as the inter-generational transfer of education has been well-established in previous literature [34]. It is possible that this college attendance could in turn provide greater opportunities for substance use [35]. However, the relationships observed in this study were found for both measures of SES, parental education and household income, indicating that these relationships were consistent across measures and not limited to parental education.

Several limitations to this study must be noted. AddHealth is an observational study, not a randomized control trial, thus causality is difficult to establish with certainty. However, the longitudinal nature of this analysis helps somewhat to address this issue. Sample loss due to attrition and item non-response is also problematic. Details of how this was addressed were discussed in the Methods section and in Additional file 1. Additionally, the outcome variables measure self-reported substance use, rather than clinically-diagnosed substance abuse or dependence. It should be noted that AddHealth is a nationally-representative sample of US secondary school students at the time of data collection, and thus does not capture adolescents who are not enrolled in school. It also does not offer extensive data on early childhood, as data collection begins when participants are in grades 7-12 at Wave I.

Despite its limitations, the AddHealth data allows for a longitudinal analysis of the relationship between SES in adolescence and subsequent substance use in early adulthood. The richness of the AddHealth data allows for the consideration of a number of facets of SES and use of a wide range of substances. It also includes a large set of individual, family, school and community characteristics assessed at baseline, including substance use and mental health at baseline. This paper contributes to the understanding of the relationship between adolescent SES and subsequent substance use, which can help educators, parents and policymakers to identify adolescents who may be at risk of substance abuse contemporaneously and in the future.

## Conclusions

This study examines the relationship between adolescent SES and subsequent substance use in early adulthood. The association varies somewhat by the type of substance used. Higher adolescent SES, as measured by parental education and household income in adolescence, is associated with higher rates of binge drinking, marijuana and cocaine use in early adulthood. No statistically significant results were found for crystal methamphetamine or other drug use.

Previous research has shown that substance use can lead to numerous problems for young adults, including difficulties in school, in the labor market, and in the criminal justice system. As much of the previous scientific literature often focuses on substance abuse in lower SES populations [1-5,17-19], it is possible that teachers and school administrators in wealthier schools may be less likely to recognize the need for substance abuse treatment programs, if the current policy focus is on lower SES populations. Likewise, administrators of drug abuse prevention programs may be less likely to focus their efforts in higher-income areas. This study offers evidence that wealthier students may be at risk for substance use problems in the future, particularly for binge drinking, marijuana and cocaine use. As previous evidence shows that students with more spending money might be more likely to engage in substance use into adulthood, access to allowances and other forms of spending money may be issues that parents can address if they are concerned with the possibility of substance abuse among their children. School administrators seeking to identify substance use education policies in their schools can find a listing of programs shown to be effective on the website for the National Institute on Drug Abuse (NIDA) [36]. Examining the substance abuse problems facing students with higher SES can help teachers, school administrators, and parents recognize the needs that may be present in their schools and communities, and the need for programs to effectively address substance use.

## Competing interests

The authors declare that they have no competing interests.

## Authors' contributions

JH is the sole author and therefore conceived and designed the study, performed statistical analysis and drafted the manuscript. This was conducted as part of a PhD dissertation.

## Supplementary Material

Additional file 1 **Missing Observations Analysis**. Description of how sample loss and attrition were examined for possible bias and precision loss [37] Click here for file
