# Supplementary material for: Are adolescents with high socioeconomic status more likely to engage in alcohol and illicit drug use in early adulthood?
Source: Subst Abuse Treat Prev Policy. 2010 Aug 5;5:19. doi: 10.1186/1747-597X-5-19 (PMC2924306; doi:10.1186/1747-597X-5-19)
Supplement: Additional file 1 — Missing Observations Analysis. Description of how sample loss and attrition were examined for possible bias and precision loss [37] [file 1747-597X-5-19-S1.DOC]

# Analysis of Missing Data

There is a sizable sample loss associated with this study, both attrition and item non-response. Of the 20,745 respondents who initially participated in the in-home interview in Wave I, only 15, 170 were able to be contacted for the Wave III interview. Additionally, 45 respondents for the genetic sample (siblings were recruited for the study who weren’t otherwise in the main sampling frame), who were only interviewed at Wave II, were contacted again in Wave III, of which 27 responded. Thus, a total of only 15,197 were contacted for the Wave III interview. In addition, a number of respondents were missing information on individual variables in the analysis.

To prevent sample loss as much as possible, information from multiple sources (parent, adolescent, baseline and Wave II) was used if available. For example, information on parental education was asked of the parent in the baseline interview, and of the adolescent in both the baseline and Wave II interviews. Thus, for parents’ education, the parent’s response was used, but if it was missing, the child’s response at baseline was used, and if still missing, the child’s response at Wave II was used (since parental education was not likely to change substantially in the one year between Waves I and II).

Finally, the adolescent’s report of general health was used, but replaced with the parent’s report of the adolescent’s general health if the adolescent report was missing. The parental report is not a perfect correlate of the child’s report. The parent and child reports of health were correlated at about 30 percent. This was the same for older adolescents (over age 16) as younger adolescents (under age 16). Although the parental report is not a perfect proxy, it is preferable than losing these cases from the sample altogether.

With these steps taken, however, there was still a fair bit of sample loss, particularly among the income and parent mental health variables, which were only asked of the parents. The final sample size was 9,872 if listwise deletion methods are used. In addressing this problem, the first step was to determine whether it was necessary to correct for both attrition and non-response or just non-response. AddHealth developed sample weights to correct for attrition up to Wave III. An initial examination showed that adolescent SES and mental health were not significantly associated with probability of inclusion in the sample once AddHealth’s weights are included.

**Reweighting**

The data were then re-weighted, to determine if bias was present due to item non-response. This was done by designing inverse probability weights (IPW) that account for the probability of selection into the sample [37]. The variables in the full model predicted inclusion in the sample (p-value on significance of the full model is <0.0001). Thus, these models were used to generate the predicted probabilities for sample selection, which were used to generate the new weights. A logit model was run, and the predicted probability of inclusion in the sample was calculated. The weights were calculated as the inverse of the predicted probabilities (A/P) where A= AddHealth’s weight, in order to account for AddHealth’s attrition correction, since it was determined earlier that AddHealth’s sampling weights effectively account for attrition. Both Pearson’s and Spearman’s correlations showed that the new weights correlated very strongly to AddHealth’s original weights: the Pearson’s correlation was 0.9861 and the Spearman’s correlation was 0.9940. Thus, any further correction for differential due to MH and SES, beyond that implicit in AddHealth’s own correction for differential sampling and sample loss, should lead to a modest change in the results.

But to verify that conjecture, the models were run using the new weights, in order to compare the results under AddHealth’s original weighting to the new weights (Appendix Table 1, first column). The results were qualitatively similar under both the original weighting scheme and under the re-weights. Because the re-weighted results are similar to those under AddHealth’s, the main analyses in the paper were reported using AddHealth’s weights, because this makes it easier to replicate this work by other researchers.

**Imputation**

The above findings indicate that there is not likely to be a substantial bias problem due to failing to correct explicitly for the sample loss as long as the sample loss is missing at random (which, as established above, can be shown when appropriate sample weights are used). However, there is still a loss of precision, which can make it more difficult to obtain a statistically significant a set of results and may make it difficult to detect subgroup differences. To address this, missing values from parental responses were imputed. Household income and parental alcoholism, both reported by parents, had the highest number of missing values. Imputation results are reported in the second column and third columns of Appendix Table 1. Imputation analysis was conducted two ways: a best-regression imputation, conducted using Stata’s impute command, and multiple regression, conducted by Stata’s ICE command.

In the best-regression imputation, household income was imputed using a linear regression equation where parental education, occupation and age were used to predict household income. Although the dependent variable in this equation, household income, is skewed (with a long tail on the right hand side of the distribution), no corrections to this (such as using the log of income) were made, since the goal of this equation was to impute actual income, rather than log income. Parental alcoholism was also imputed using a linear probability model, where the adolescent’s CES-D score and substance use (number of substances used), as well as the adolescent’s perception of the relationship to the parents, was used to predict parental alcoholism. Imputation flags were then created, to identify whether the imputed values were significantly associated with the outcome. If the flags are not significant, this indicates that observations that were missing values on the given item are not substantially influencing the outcome. Interactions were also created between the key mental health and SES variables and the imputation flag. A significant interaction term in this case would indicate that the relationship between the mental health and/or SES variables and the outcome is different for observations with missing information on income and parental mental health than for those not missing this information.

The second column of Appendix Table 1 shows the results with household income and parental mental health imputed. The non-imputed results are also presented for comparison in the fourth column. Overall, the results are similar to the non-imputed results. The imputation flags for household income and parental alcoholism are not statistically significant, indicating that the imputation is not correlated with the outcome.

Finally, a multiple imputation analysis was conducted (using Stata’s ICE command). Household income, parental education, and parent alcoholism were imputed using these variables along with the parent’s age and occupation and the adolescent’s CES-D score, AOD use and whether they report feeling close to their parents. This generated a sample size of 13,418. Imputation results are reported in the third column of Appendix Table 1. Results are similar to the best-regression imputation. As imputed results do not differ substantially from the original sample, the non-imputed AddHealth sample was used in the main analyses to allow for greater replication.

**Appendix Table 1. Logistic Regression Results under Reweighting, Imputation, and Original Results Predicting Alcohol Use in Wave III**

|  | Reweighting  (n=9872) | Best-regression Imputation  (n=13,328) | Multiple Imputation  (n=13,418) | Original Results  (n=9456) |
| --- | --- | --- | --- | --- |
| Parent Alcoholism | 1.046  (0.838-1.304) | 1.006  (0.819-1.235) | 0.999  (0.802-1.242) | 1.015  (0.818-1.259) |
| Household Income (in thousands) | 1.003  (1.001-1.004)*** | 1.003  (1.002-1.098)*** | 1.002  (1.001-1.004)** | 1.003  (1.001-1.004)*** |
| Income Impute Flag | -- | 0.789  (0.567-1.098) | -- | -- |
| Parent Alcohol Impute Flag | -- | 1.110  (0.731-1.687) | -- | -- |

Note: Dependent variables assessed at Wave III. Independent variables assessed at Wave I. All models control for binge drinking, marijuana use, cocaine use, inhalant use and other drug use at baseline, other mental health at baseline (CESD, delinquency, suicidality PPVT score, gender, age, age squared, race/ethnicity, general health, family structure (two biological parents, single parent, stepfamily, foster/other), parent alcoholism and whether adolescent is firstborn in family. Best regression imputation model also includes interactions between imputation flags for income and parental alcoholism and adolescent’s mental health and substance use at baseline.

* p<0.10, ** p<0.05, *** p<0.01
